# Genetic toxicity testing using human in vitro organotypic airway cultures: Assessing DNA damage with the CometChip and mutagenesis by Duplex Sequencing

**DOI:** 10.1002/em.22444

**Published:** 2021-06-14

**Authors:** Yiying Wang, Roberta A. Mittelstaedt, Rebecca Wynne, Ying Chen, Xuefei Cao, Levan Muskhelishvili, Kelly Davis, Timothy W. Robison, Wei Sun, Elizabeth K. Schmidt, Thomas H. Smith, Zachary K. Norgaard, Charles C. Valentine, Jeffry Yaplee, Lindsey N. Williams, Jesse J. Salk, Robert H. Heflich

**Affiliations:** ^1^ U.S. Food and Drug Administration, National Center for Toxicological Research Jefferson Arkansas USA; ^2^ Toxicologic Pathology Associates Jefferson Arkansas USA; ^3^ U.S. Food and Drug Administration, Center for Drug Evaluation and Research Silver Spring Maryland USA; ^4^ Twinstrand Biosciences, Inc. Seattle Washington USA

**Keywords:** DNA damage, error‐corrected next generation sequencing (ecNGS), ethyl methanesulfonate (EMS), human in vitro air‐liquid‐interface (ALI) airway epithelial tissue model, mutagenesis

## Abstract

The organotypic human air‐liquid‐interface (ALI) airway tissue model has been used as an in vitro cell culture system for evaluating the toxicity of inhaled substances. ALI airway cultures are highly differentiated, which has made it challenging to evaluate genetic toxicology endpoints. In the current study, we assayed DNA damage with the high‐throughput CometChip assay and quantified mutagenesis with Duplex Sequencing, an error‐corrected next‐generation sequencing method capable of detecting a single mutation per 10^7^ base pairs. Fully differentiated human ALI airway cultures were treated from the basolateral side with 6.25 to 100 μg/mL ethyl methanesulfonate (EMS) over a period of 28 days. CometChip assays were conducted after 3 and 28 days of treatment, and Duplex Sequencing after 28 days of treatment. Treating the airway cultures with EMS resulted in time‐ and concentration‐dependent increases in DNA damage and a concentration‐dependent increase in mutant frequency. The mutations observed in the EMS‐treated cultures were predominantly C → T transitions and exhibited a unique trinucleotide signature relative to the negative control. Measurement of physiological endpoints indicated that the EMS treatments had no effect on anti‐p63‐positive basal cell frequency, but produced concentration‐responsive increases in cytotoxicity and perturbations in cell morphology, along with concentration‐responsive decreases in culture viability, goblet cell and anti‐Ki67‐positive proliferating cell frequency, cilia beating frequency, and mucin secretion. The results indicate that a unified 28‐day study can be used to measure several important safety endpoints in physiologically relevant human in vitro ALI airway cultures, including DNA damage, mutagenicity, and tissue‐specific general toxicity.

## 1 INTRODUCTION

Organotypic in vitro tissue models are anticipated to create unique opportunities to improve predictive toxicology, reduce animal testing, and advance regulatory science (FDA, 2017). Potentially, these models can be used to evaluate the effects of chemicals on relevant physiological processes in a cost‐ and time‐effective manner, and without the need for conducting animal experiments. If organotypic in vitro model systems are validated, and appropriate in vitro to in vivo extrapolation methods developed, the models could wholly replace rather than just supplement animal data in drug and chemical risk assessments.

One area where in vitro tissue models can have a major impact is in evaluating the toxicity of inhaled substances. Rodent inhalation toxicology studies are extremely resource intensive. In addition, the data from rodent inhalation exposures can be difficult to interpret in the context of human risk assessment because of differences in the architecture and biochemistry of rodent and human airways (Chamanza & Wright, 2015; Hofmann et al., 1989). Human primary bronchial epithelial cell‐based in vitro tissue models and ex vivo models employing human airway tissue slices have been used to study inhalation toxicology for many years. However, appropriate characterization, validation, and qualification of these models are required for performing regulatory toxicity assessments (Cao et al., 2020; Lacroix et al., 2018).

The human in vitro air‐liquid interface (ALI) airway epithelial tissue model has been of particular interest for inclusion into a robust in vitro inhalation toxicology platform. The ALI tissue model is composed of the major cell types that populate the human lower large airway, including beating ciliated cells, mucus‐producing goblet cells, and progenitor basal cells, and the model retains many of the structural and functional characteristics of the intact human airway epithelial tissue from which it is derived (BéruBé et al., 2009; Cao et al., 2015; Cao et al., 2020). The cells in these models are oriented in a polar fashion, with the upper (apical) surface being exposed to air and covered with beating cilia and patches of mucus. The bottom (basolateral) surface is attached to a microporous membrane that is bathed by medium from below to provide nutrients to the culture. Besides their physical and functional similarity to the natural lining of the human airway, the air interface permits the exposure of these models to aerosols, smoke, and gases in a manner similar to human inhalation exposures (Cao et al., 2020). A number of disease‐relevant physiological and molecular tissue responses have been measured in human ALI airway cultures (Cao et al., 2015; Xiong et al., 2018). Evaluation of these endpoints is expected to increase the value of in vitro toxicology studies for regulatory decision‐making.

The measurement of genotoxicity in somatic tissues, including the airway, is of importance for evaluating the carcinogenic potential of inhaled substances and the regulatory safety assessment of drugs and chemicals. Genotoxicity assessment, however, is challenging for many in vitro organotypic tissue models because they are predominately composed of highly differentiated, non‐dividing cells (Pfuhler et al., 2020). In the case of human ALI airway cultures, only about 5% of the cells stain with anti‐Ki67 antibodies, meaning that very few of the cells are dividing and thus capable of converting DNA damage into stable, inheritable DNA sequence changes (i.e., mutations). In a recent review of the field conducted by the International Workshops on Genotoxicity Testing, van Acker reported on their attempts to adapt a micronucleus assay to the human airway model and found that the number of binucleated cells generated in the cultures were too few to assay for genetic damage (Pfuhler et al., 2020). To date, the majority of genotoxicity assessments in these cultures have been limited to monitoring DNA damage with the comet assay (Pfuhler et al., 2020; Qin et al., 2019).

In this current, proof‐of‐principle study, we applied two recently developed tools to measure genotoxicity endpoints in an ALI airway tissue model exposed to the prototypical mutagen, ethyl methanesulfonate (EMS). We employed a 28‐day exposure protocol, which is recommended for measuring mutagenesis in vivo in the transgenic rodent assay (OECD, 2020), because this organotypic model has cell‐cycle kinetics similar to those found in vivo. We anchored our study by measuring a genotoxicity endpoint that was successfully monitored previously in the ALI airway model, i.e., DNA damage by the comet assay. In our current study, however, we used the high‐throughput CometChip assay (Sykora et al., 2018; Wood et al., 2010). For measuring mutagenesis, we anticipated that standard methods for measuring gene mutation based on phenotypic selection (such as measuring mutation in the hypoxanthine‐guanine phosphoribosyltransferase (*HPRT*) or thymidine kinase (*TK*) genes) or cell surface markers (such as measuring *PIG‐A* mutation) would be difficult, requiring generating single cells by tissue disaggregation and perhaps expansion of the single cells into clones (Kucab et al., 2019). Forming single cell clones likely would be extremely inefficient in a tissue model consisting largely of terminally differentiated cells. Using whole genome sequencing to identify mutations in the bulk cell population would be impossible because the error rate of such methods are orders of magnitude higher than the frequency of induced mutations (Salk et al., 2018). To circumvent these potential hurdles, we used Duplex Sequencing, a DNA‐based error‐corrected next‐generation sequencing (ecNGS) approach, for measuring mutations in these cultures. Duplex Sequencing has an error rate below one in ten‐million and is capable of directly assessing the frequency and pattern of mutations distributed across the genome (Salk et al., 2018; Salk & Kennedy, 2020; Valentine et al., 2020). Based on compelling observations with the ALI airway model in this study, we anticipate that the CometChip and ecNGS Duplex Sequencing assays will find applications for measuring genotoxicity in other highly differentiated tissue models as these systems become increasingly used for advanced, next generation toxicology assessments.

## 2 MATERIALS AND METHODS

### 2.1 Human ALI airway tissue model

**FIGURE 1:**
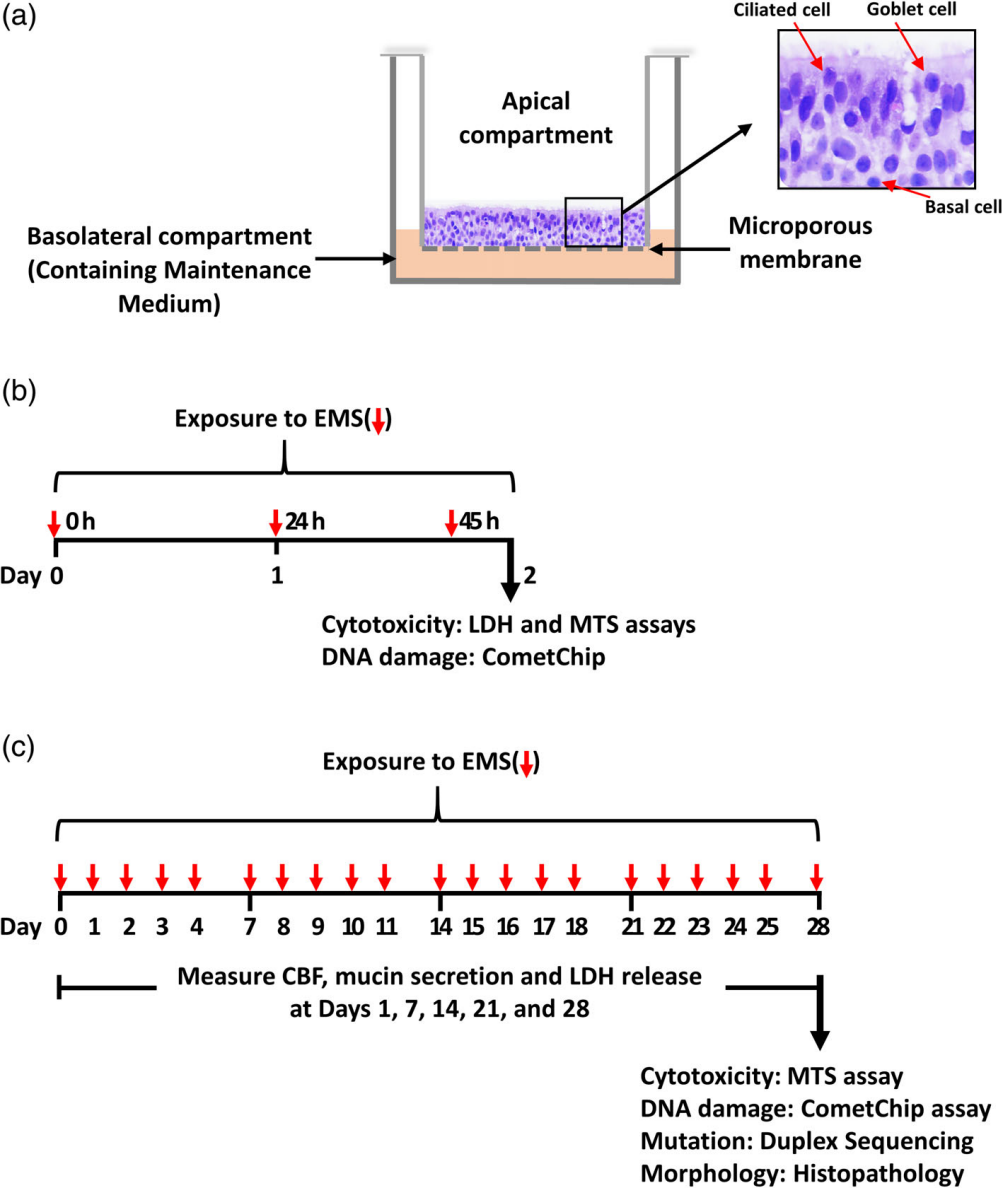
Schematic diagrams of the human ALI airway culture, and experimental design. (a) Shows an image of an H&E stained transverse section from a human ALI tissue model that was used in this study. A microporous membrane that provides the permeable support for cell growth, divides the culture plate well into apical (upper) and basolateral (lower) compartments. The human ALI airway culture consists of ciliated cells, goblet cells, and basal cells. The apical side of the cultures is exposed to the air and Maintenance Medium is added to the basolateral compartment. The cultures were treated with various concentrations of EMS for periods of (b) 3‐days or (c) 28‐days. LDH release, cilia beating frequency and mucin secretion were monitored at Day 1, 7, 14, 21 and 28 of the 28‐day treatment period in (c). The CometChip and cell viability assays were conducted following the 3‐day treatment in (b) and the 28‐day treatment in (c). Cultures were collected for Duplex Sequencing or for morphological investigation at Day 28 in (c). Shorter red arrows: dosed medium added at 0, 24 and 45 h in (b) and Monday through Friday for 28 days in (c); longer arrows: assays performed 3 h after the last treatment (b and c). LDH, lactate dehydrogenase; CBF, cilia beating frequency

Human ALI airway tissue models (Figure 1(a)) were generated as previously described (Cao et al., 2018). Briefly, human primary tracheobronchial epithelial cells (MatTek, Ashland, MA) were expanded in PneumaCult™‐Ex Medium (STEMCELL Technologies, Seattle, WA) and 4 × 10^4^ cells were seeded onto each 24‐well PET Transwell® cell culture insert (Corning, Corning, NY). When 100% confluence was reached, PneumaCult™‐ALI Maintenance Medium (STEMCELL Technologies) was added only to the basolateral side of the insert, with the apical surface of the cultures exposed to air, a step referred to as “air‐lifting”. Four weeks post air‐lift, the airway cultures were fully differentiated and ready for use in experiments.

### 2.2 Exposure of ALI airway cultures to EMS

Ethyl methanesulfonate (EMS; Sigma–Aldrich, St. Louis, MO) was dissolved in dimethylsulfoxide (DMSO, Sigma–Aldrich) and serially diluted to 100× working concentrations of 0.625, 2.5, 5.0 and 10 mg EMS/mL immediately before use. Preliminary concentration and time range‐finding studies were conducted to determine a maximum concentration tolerated by airway cultures and to establish the treatment regimen. For treatments, the working solutions were diluted 1:100 (vol/vol) in the PneumaCult™‐ALI Maintenance Medium to achieve final concentrations of 6.25, 25, 50 and 100 μg EMS/mL; DMSO vehicle only was added to the treatment medium for the negative control. For the 3‐day treatment, cultures were exposed from the basolateral side by feeding the cultures with Maintenance Medium containing DMSO or various concentrations of EMS, with the exposure medium changed three times at 0, 24 and 45 h for a total of 48 h of exposure (Figure 1(a),(b)). For the 28‐day treatments, cultures were treated with EMS or DMSO from the basolateral side over a 28‐day period, with treatment medium refreshed daily on Monday through Friday (Figure 1(a),(c)).

Non‐tissue‐perturbing endpoints, including lactate dehydrogenase (LDH) release, cilia beating frequency (CBF), and mucin secretion, were evaluated every 7 days over the 28‐day treatment period. For the CometChip and MTS cell viability assays, cells were dissociated into single cells by 0.25% trypsin (Thermo Fisher Scientific, Gibco™, Waltham, MA) 3 h after the 3rd and the final treatments. After 28 days of treatment, the airway cultures also were collected as cell pellets for DNA extraction and subsequent Duplex Sequencing or fixed as intact cell cultures in 10% neutral‐buffered formalin for morphological and immunohistochemical assessment (Figure 1(c)).

### 2.3 LDH cytotoxicity assay

The cytotoxicity caused by EMS in ALI airway cultures was assessed by measuring LDH release into the basolateral medium using a Lactate Dehydrogenase (LDH) Activity Assay Kit (Roche, Indianapolis, IN). The reaction mixture was freshly prepared by diluting the kit‐supplied catalyst in dye solution at a ratio of 1:45 (v/v) and 100 μL of the reaction mixture were incubated with 100 μL of basolateral medium for 15 min at room temperature in the dark. Fifty μL of stop solution were added to terminate the reactions. Absorbance was measured at 490 nm using a Synergy H4 microplate reader (BioTek, Winooski, VT).

### 2.4 Cilia beating frequency (CBF)

Cultures were placed on a heated microscope stage and equilibrated to 30°C before CBF measurement. Cilia motility was recorded using a high‐speed camera (Ammons Engineering, Clio, MI) connected to a Leica DMI4000B microscope (Buffalo Grove, IL). CBF was analyzed for each culture in four fields free of mucus clumps using the Sisson‐Ammons Video Analysis System (SAVA System, Ammons Engineering).

### 2.5 Mucin secretion

Mucin secretion was measured in apical washes by ELISA, as described previously (Xiong et al., 2018). Washes were collected by bathing the apical (cellular) side of the cultures twice with 100 μL Dulbecco's Phosphate‐Buffered Saline (DPBS, pH 7.4; Corning, Manassas, VA) containing 0.025 mM dithiothreitol. Cell debris was removed from the washes by centrifugation at 600*g* for 10 min at 4°C.

Fifty μL of the apical washes were coated onto a high‐protein‐binding ELISA plate (Thermo Fisher Scientific) for mucin quantification. The ELISA plate was blocked with 5 mg/mL bovine serum albumin (BSA) diluted in DPBS for 1 h at room temperature, followed by incubation for 1 h with primary antibodies (mouse anti‐MUC5AC, Pierce, Rockford, IL, catalog no. MA5‐12178; mouse anti‐MUC5B, Abcam, Cambridge, MA, catalog no. ab779955) diluted at 1:500 in DPBS containing 5 mg/mL BSA, 0.15% Triton X‐100, and 0.1% Tween‐20. Subsequently, 100 μL horseradish‐peroxidase‐conjugated goat‐anti‐mouse antibody (Rockland, Limerick, PA) diluted at 1:5000 in DPBS containing 5 mg/mL BSA, 0.15% Triton X‐100, and 0.1% Tween‐20 were added to each well, followed by a 1‐h incubation. Excess antibodies were removed by washing the plates three times with DPBS. Color was developed by adding 50 μL 3,3′,5,5′‐tetramethylbenzidine (Thermo Fisher Scientific) and the reactions were stopped with an equal volume of 2 N HCl. The absorbance at 450 nm was measured using a Synergy H4 microplate reader.

### 2.6 MTS cell viability assay

Three hours following EMS exposure on Day 3 and Day 28, cells from each 24‐well cell culture insert were dissociated with 0.25% trypsin and resuspended in 600 μL of ice‐cold DPBS as a single cell suspension and were analyzed with the MTS cell viability assay and CometChip assay. The CellTiter® 96 Aqueous Nonradioactive Cell Proliferation MTS Assay (Promega, Madison, WI) assesses metabolic conversion of a tetrazolium compound, 3‐(4,5‐dimethylthiazol‐2‐yl)‐5‐(3‐carboxymethoxyphenyl)‐2‐(4‐sulfophenyl)‐2H‐tetrazolium (MTS), to a soluble formazan product in live cells, as a quantitative indicator of cell viability. Eighty μL of the cell suspension were mixed with 120 μL of fresh Maintenance Medium containing 1 × MTS/PMS reagent. The mixture was incubated for 1 h at 37°C. The formazan dye was quantified by measuring absorbance at 490 nm using a Synergy H4 plate reader.

### 2.7 CometChip assay

The remaining cell suspension prepared as described for the MTS cell viability assay was used to perform the CometChip assay according to the manufacturer's alkaline assay protocol (Trevigen, Gaithersburg, MD) with some modifications. One hundred μL of the suspension were transferred to each of 5 wells of a 96‐well CometChip (Trevigen), with each well containing approximately 400 microwells. The cells were allowed to settle by gravity into the microwells for 30 min at room temperature. After cell loading, the CometChip was rinsed gently with 25 mL DPBS and then sealed with low melting point agarose (Trevigen). The cells then were treated with lysis solution (Trevigen) overnight at 4°C. DNA unwinding and electrophoresis were performed according to the Trevigen protocol. The CometChips then were neutralized in 0.4 M Tris–HCl buffer (pH 7.4, Sigma‐Aldrich) and equilibrated in 0.02 M Tris–HCl buffer (pH 7.4). Subsequently, the DNA was stained with 0.2 ×  SYBR® Gold (Invitrogen, Carlsbad, CA) overnight at 4°C and images were acquired using a BioTek Cytation 5 Image Reader (BioTek, Winooski, VT). Trevigen Comet Analysis Software was used to score the percentage of DNA in the comet tail (%DNA in Tail) for at least 300 cells from each culture insert.

### 2.8 Duplex Sequencing

Genomic DNA was extracted from cell pellets using a QIAamp DNA Mini Kit (QIAGEN, Valencia, CA) following the manufacturer's suggested protocol. DNA concentration was measured using an Invitrogen™ Qubit™ dsDNA HS Assay Kit (Thermo Fisher Scientific).

Duplex Sequencing was performed as described previously, with minor modifications (Valentine et al., 2020), using the TwinStrand Duplex Sequencing™ Human Mutagenesis kit according to the manufacturer's protocol (Rev 1.0; TwinStrand Biosciences, Seattle, WA). Briefly, 400 ng of DNA were ultrasonically sheared, end‐repaired, A‐tailed, ligated with Duplex Adapters and treated with a kit‐supplied conditioning enzyme cocktail. Each library was amplified using primers containing unique dual‐index sequences and then subjected to hybrid capture with the Human‐50 Mutagenesis Panel (Rev. 1.0; TwinStrand Biosciences). The panel consisted of an optimized set of genome‐representative DNA sequences in twenty 2.4‐kb loci not believed to be under significant negative or positive selection pressure and spread across the autosomes of the human genome. Indexed libraries were pooled and sequenced using 151 base pair paired‐end reads on an Illumina NovaSeq 6000 or NextSeq 550 (Illumina, San Diego, CA).

Primary data analysis was carried out on a cloud‐compute system using TwinStrand software. Briefly, sequencing reads were aligned to the hs38DH reference sequence (ftp://ftp.ncbi.nlm.nih.gov/genbank/genomes/Eukaryotes/vertebrates_mammals/Homo_sapiens/GRCh38/seqs_for_alignment_pipelines/GCA_000001405.15_GRCh38_full_analysis_set.fna.gz) (Jäger et al., 2016). Reads sharing alignment coordinates and a common molecular tag in both strand orientations were grouped and assembled into error‐corrected duplex consensus sequences and re‐aligned to the hs38DH reference. Libraries generated a mean on‐target depth of 12,785× (range 6786× to 16,724×), producing a total of 13.5 billion error‐corrected nucleotides (Table 1). Because bases in duplex consensus alignments correspond to original double‐stranded DNA molecule nucleotides, the number of non‐ambiguous duplex consensus bases reflects the number of nucleotides in the original source molecules from the ALI cultures. For each sample, the mutant frequency was calculated as the number of unique mutations (defined as variant bases with a variant allele frequency below 1% to exclude inherited polymorphisms) divided by the total number of non‐ambiguous duplex consensus bases sequenced within the panel's territory.

**TABLE 1:** Quantitative data describing duplex sequencing mutation analysis of EMS‐treated human ALI airway tissue models

Treatment	Replicate	Total duplex bases sequenced (×10^6^)	Mutant nucleotides detected	Mutant frequency (×10^−7^)
Vehicle control	1	481	76	1.58
	2	513	75	1.46
	3	636	156	2.45
	4	727	131	1.80
**Mean**		**589**	**110**	**1.86**
6.25 μg/mL	1	796	189	2.37
	2	323	64	1.98
	3	709	174	2.45
**Mean**		**609**	**142**	**2.27**
25 μg/mL	1	699	220	3.15
	2	726	244	3.36
	3	725	205	2.83
**Mean**		**717**	**223**	**3.11**
50 μg/mL	1	444	161	3.62
	2	456	172	3.77
	3	685	300	4.38
	4	642	280	4.36
**Mean**		**557**	**228**	**4.03**
100 μg/mL	1	423	423	10.00
	2	398	325	8.17
	3	746	875	11.73
	4	407	458	11.24
**Mean**		**494**	**520**	**10.28**

### 2.9 Histology

After one wash with DPBS, cell culture inserts were fixed in 10% neutral‐buffered formalin for 48 h, routinely processed into paraffin‐embedded blocks, sectioned to a thickness of 4–5 μm, and applied to positive‐charged glass slides. The sections were then deparaffinized in xylene, rehydrated through a graded series of ethanol to distilled water, and stained with hematoxylin–eosin (H&E) and by periodic acid‐Schiff (PAS). Morphology and PAS‐stained goblet cells were assessed visually by light microscopy (BX40, Olympus, Tokyo, Japan).

### 2.10 Immunohistochemistry (IHC)

For immunohistochemical detection of Ki67 and p63 expression, slides were placed in an antigen retrieval solution (0.01 M citrate buffer, pH 6.0) for 15 min in a microwave oven set at 100°C and 800 W. Slides then were stained using an Autostainer 360 (Thermo Fisher Scientific). Tissue sections were incubated in 3% hydrogen peroxide for 10 min to inactivate the endogenous peroxidases. Nonspecific staining was blocked using 10% goat serum (anti‐Ki67) or 10% rat serum (anti‐p63) for 20 min. The tissue sections then were incubated with primary antibodies anti‐Ki67 (rabbit monoclonal, Thermo Fisher Scientific, catalog no. RM‐9106) or anti‐p63 (mouse monoclonal, Biocare Medical, Pacheco, CA, catalog no. CM163C) for 1 h at room temperature at a dilution of 1:100, followed by incubation with biotinylated goat anti‐rabbit or rat anti‐mouse secondary antibodies (diluted 1:200; Jackson ImmunoResearch Laboratories, West Grove, PA) for 30 min at room temperature. Slides then were incubated in ExtrAvidin peroxidase (1:100, Sigma‐Aldrich). Positively stained cells were visualized following treatment with 3′‐diaminobenzine (DAB) for 5 min at room temperature. Sections were counter‐stained with hematoxylin, dehydrated, cleared in xylene, and mounted with Permount™ Mounting Medium prior to microscope visualization.

### 2.11 Image analysis

IHC‐ or PAS‐stained sections were scanned and digital images were obtained with the Aperio Scanscope System (Leica Biosystems, Vista, CA). The percentage of anti‐Ki67‐ or anti‐p63‐positive nuclei was calculated using the Nuclear Algorithm (Aperio). Goblet cell density (%) was evaluated semi‐automatically. The total numbers of nuclei were counted with the Nuclear Algorithm, while PAS‐stained goblet cells were counted manually.

### 2.12 Statistical analyses

Most data are presented as the mean ± SD of replicate observations. For mutation data, confidence intervals for point estimates of mutant frequency were set to the 95% threshold and were computed with the Wilson method. Statistical differences between the vehicle control and EMS‐treated groups were analyzed by one‐way analysis of variance (ANOVA) followed by Dunnett's test using SigmaPlot 13.0 (Systat Software, San Jose, CA). Comparisons having *p*‐values ≤ .05 were considered significant.

## 3 RESULTS

### 3.1 Cytotoxicity of EMS in ALI airway cultures

**FIGURE 2:**
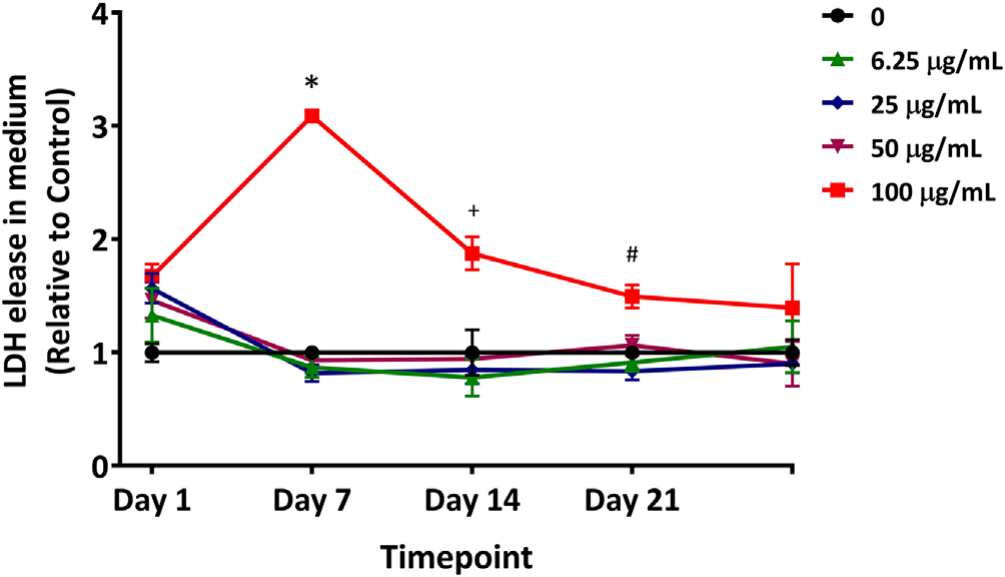
Cytotoxicity in EMS‐treated ALI airway cultures. Cytotoxicity was evaluated using the LDH assay in cultures used for Duplex Sequencing. Data presented as means ± SD (*n* = 3). *^,+,#^ *p* < .05 was considered statistically significant when compared to concurrent vehicle controls; different symbols refer to comparisons made to controls at different time points. A table with primary data has been included as Supporting Information

The toxicity of the EMS treatment was assessed by measuring the release of LDH from the ALI cultures into the basolateral medium on Day 1, 7, 14, 21, and 28 (Figure 2). Concentrations up to 50 μg/mL EMS did not significantly increase LDH release at any of the time points. Treatment with 100 μg/mL EMS for 7 days caused a significant increase in LDH release. Thereafter, the levels of LDH release decreased over time and were comparable to that of the control group by Day 28.

### 3.2 Effects of EMS on organotypic functions

**FIGURE 3:**
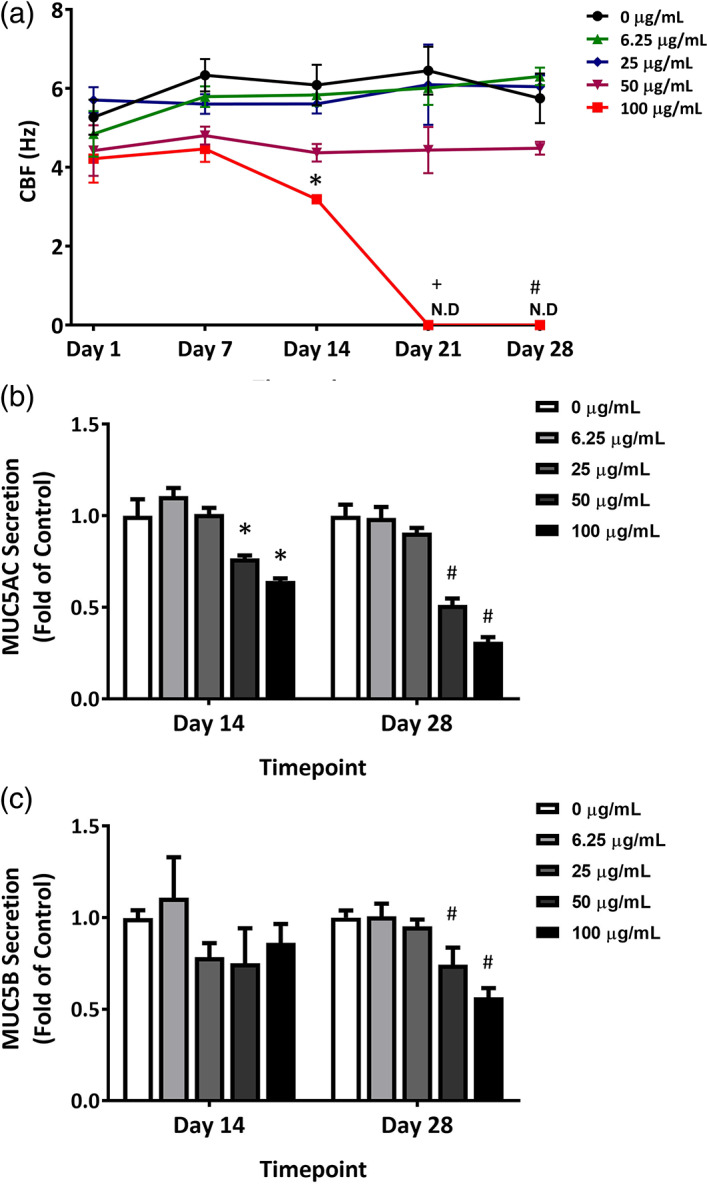
Functional changes in EMS‐treated ALI airway cultures. (a) Cilia beating frequency (CBF; *n* = 4) and (b and c) mucin secretion (*n* = 4) were measured in cultures used for Duplex Sequencing assays. Data presented as means ± SD. *^,+,#^ *p* < .05 was considered statistically significant when compared to the concurrent vehicle controls; different symbols refer to comparisons made to controls at different time points. A table with primary data has been included as Supporting Information

Mucociliary clearance (MCC) is a primary function of airway epithelium, playing a major role in its innate defense against various types of toxicants (Mall, 2008). Motile cilia on ciliated cells and mucus secretion by goblet cells, the two primary components of MCC, were monitored as indirect indicators of the adverse effect of EMS on the airway cultures. CBF was measured after 1, 7, 14, 21, and 28 days of treatment (Figure 3(a)). No measurable changes in CBF were observed at concentrations of 50 μg/mL and below. Treatment with 100 μg/mL EMS induced a time‐dependent decrease in CBF. The decrease in CBF occurred in cultures treated for 14 or more days; CBF was undetectable at Day 21 and 28.

The abundance of two major airway mucins, MUC5AC and MUC5B, was measured in secretions collected after 14 days and 28 days of EMS treatment. EMS exposure resulted in time‐ and concentration‐dependent decreases in mucin secretion (Figure 3(b),(c)). Inhibition of MUC5AC secretion was observed at Day 14 and 28 (Figure 3(b)). Decreases in MUC5B secretion were observed at Day 28 in cultures treated with 50 and 100 μg/mL EMS (Figure 3(c)).

### 3.3 Effects of EMS on tissue structure, goblet cell density, and immunohistochemical markers

**FIGURE 4:**
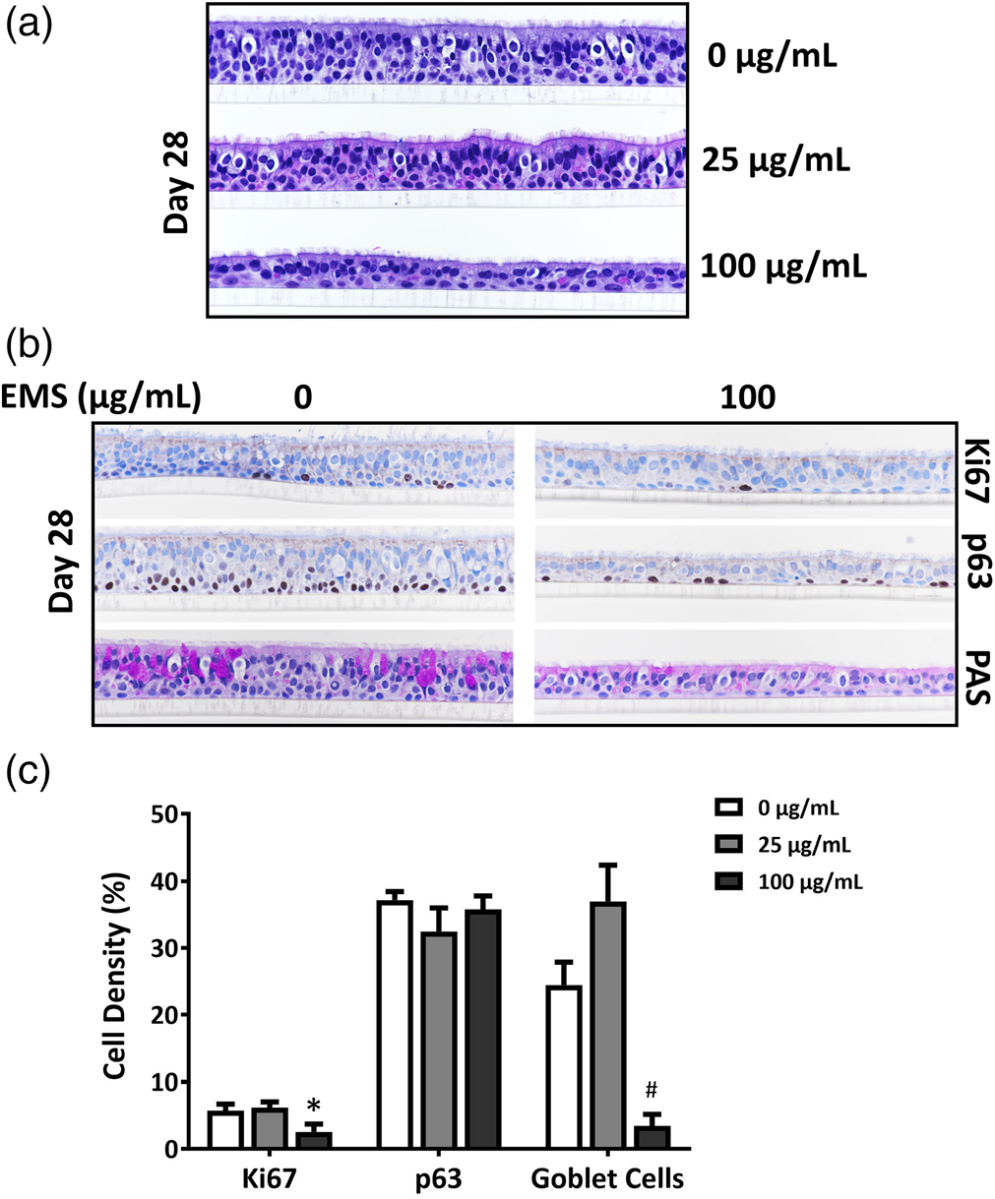
Histological and immunohistochemical (IHC) observations in EMS‐treated ALI airway cultures. (a) Shows representative images of H&E‐stained ALI airway cultures after a 28‐day treatment with EMS. Magnification is 40×. (b) Shows representative images of ALI airway cultures treated with EMS for 28 days and stained for histological and IHC observations. Magnification is 40×. Cell proliferation was evaluated by IHC with anti‐Ki67 antibody; basal cells were stained with anti‐p63 antibody and goblet cells were stained using periodic acid Schiff (PAS). (c) Shows percentages of PAS‐positive goblet cells, anti‐p63‐positive basal cells and anti‐Ki67‐positive proliferating cells. Data (*n* = 3) presented as means ± SD. *^,#^ *p* < .05 was considered statistically significant compared to the concurrent vehicle controls; different symbols refer to comparisons made to different controls. A table with primary data has been included as Supporting Information

The overall morphology of the cultures was assessed following 28 days of EMS exposure by microscope evaluation of H&E‐stained tissue sections. While treating the cultures with 25 μg/mL EMS for 28 days had minimal effects on cytotoxicity as measured with the LDH assay (Figure 2), there was visual evidence of airway epithelium atrophy which was characterized by a decrease in the thickness of the airway cultures due to a decrease in cell numbers and a decrease in cell height compared to concurrent vehicle controls (Figure 4(a)). Exposure to 100 μg/mL EMS led to more severe degeneration (Figure 4(a)). Exposing cultures to 25 μg/mL EMS had no significant effect on the density of PAS‐stained goblet cells, but treatment with 100 μg/mL EMS for 28 days resulted in near‐total depletion of goblet cells and an increase in the number of epithelial cells with no cilia, fewer cilia, or cilia of decreased height at the apical surface compared to matched vehicle controls (Figure 4(b),(c)).

As cell proliferation is necessary for the fixation of mutations, we evaluated changes in the number of cells positive for p63 (a marker of epithelial basal cells) (Rock et al., 2010) and Ki67 (a marker of cell proliferation) (Sun & Kaufman, 2018) using IHC staining with marker‐specific antibodies (Figure 4(b),(c)). No change in the percentage of anti‐p63‐positive basal cells was observed in cultures exposed to either 25 or 100 μg/mL EMS for 28 days, but the percentage of anti‐Ki67‐positive cells was significantly decreased after a 28‐day treatment with 100 μg/mL EMS.

### 3.4 EMS‐induced DNA damage detected using the CometChip assay

**FIGURE 5:**
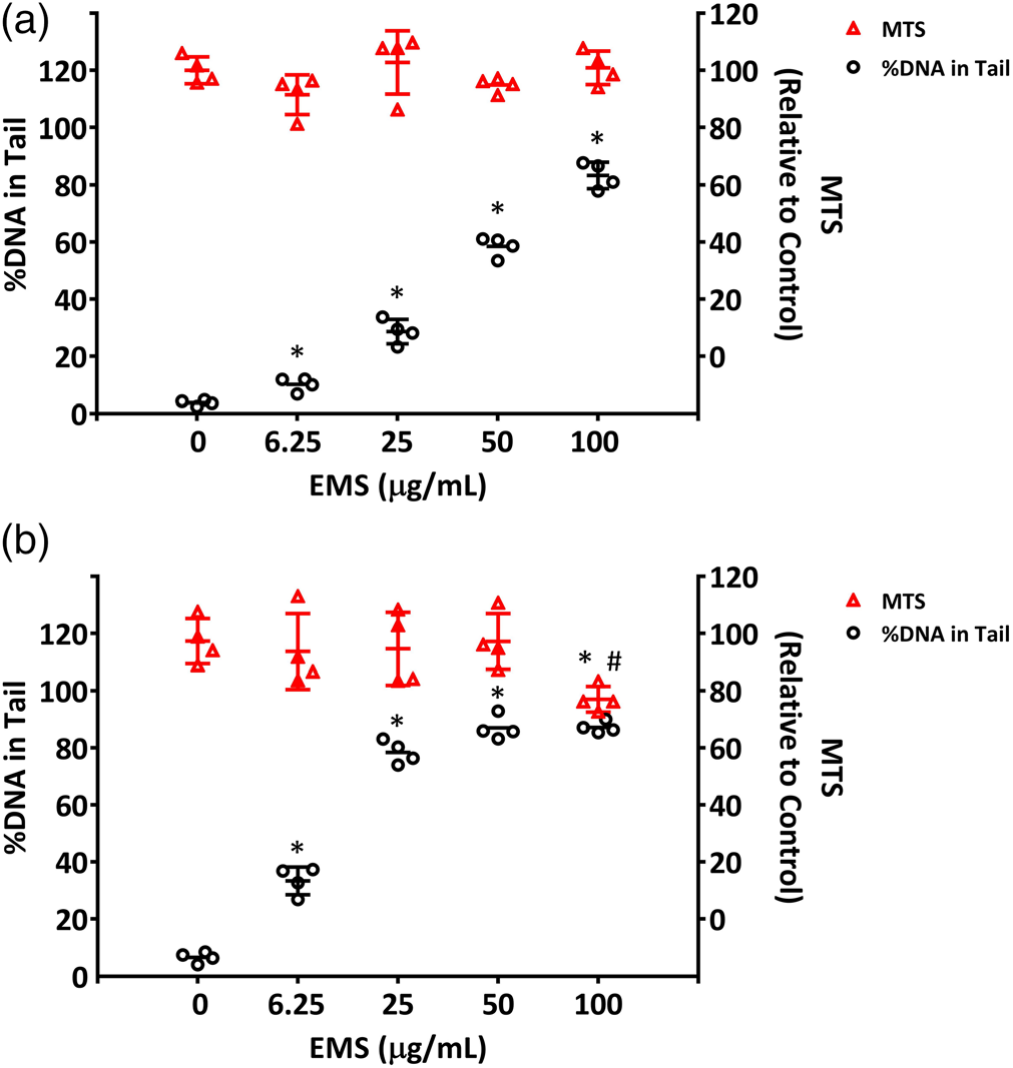
DNA damage in EMS‐treated ALI airway cultures. DNA damage (DNA strand breaks and alkali‐labile sites measured as %DNA in Tail) was detected using the CometChip assay and the relative cell viability (% of control) was measured using the MTS assay after (a) 3‐day and (b) 28‐day treatments. Values for %DNA in Tail for individual cultures (*n* = 4) (black hollow circles) are plotted on the left Y‐axis, with the mean ± SD shown; corresponding values for relative cell viability (% of control) (red squares) are plotted on the right Y‐axis, along with their mean ± SD. To promote their visualization, the results for different treatment concentrations are spaced evenly along the X‐axis, without regard to relative concentration. *^,#^ *p* < .05 was considered statistically significant compared to the respective vehicle controls; different symbols refer to comparisons made using different assays. A table with primary data has been included as Supporting Information

EMS is a direct‐acting mutagen, producing ethyl‐DNA adducts that result in clastogenicity and point mutations; some of these adducts can cause DNA strand breaks and alkali‐sensitive sites that can be detected with the comet assay (Müller et al., 2009; Pozniak et al., 2009). We found that treatment of ALI airway cultures with EMS resulted in both time‐ and concentration‐dependent increases in %DNA in Tail. The 3‐day treatment with 0, 6.25, 25, 50, and 100 μg/mL EMS resulted in 3.8, 10.2, 28.7, 58.5, and 83.3 %DNA in Tail, respectively (slope of linear regression line was significantly non‐zero, *p* = .004, Figure 5(a)). Treatment with EMS for 28 days also increased the level of %DNA in Tail in a concentration‐responsive manner, with average values for %DNA in Tail of 6.7, 33.5, 78.5, 87.0 and 87.2 for 0, 6.25, 25, 50, and 100 μg/mL EMS (slope of linear regression line was significantly non‐zero, *p* = .029, Figure 5(b)). The extent of DNA damage measured by the assay appeared to saturate at %DNA in Tail values of 80%–90%. When compared to the levels of DNA damage elicited by the 3‐day treatment (Figure 5(a)), the magnitude of responses at Day 28 suggested that DNA damage continued to accumulate with time over the 28‐day treatment period. In addition, the DNA damage was unlikely to result from EMS‐induced cytotoxicity. As measured by the MTS assay, the highest concentration of 100 μg/mL EMS had no effect on cell viability after a 3‐day treatment, whereas 100 μg/mL EMS reduced viability by only 20% after 28 days of treatment (Figure 5(a),(b)).

### 3.5 EMS‐induced mutagenesis measured by Duplex‐Sequencing

**FIGURE 6:**
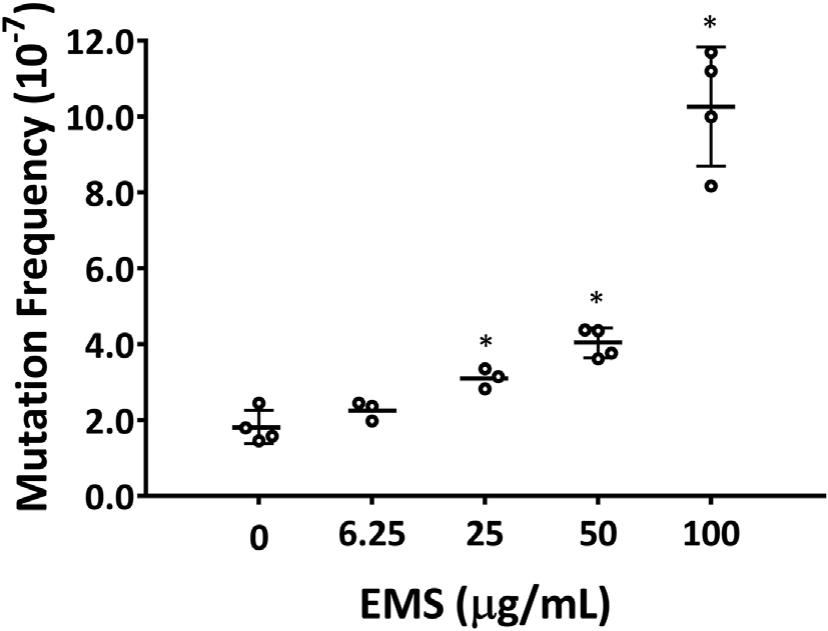
Mutant frequency in EMS‐treated ALI airway cultures. Mutation was measured using Duplex Sequencing after a 28‐day treatment with EMS. The individual data (*n* = 4, for control, 50 μg/mL and 100 μg/mL; *n* = 3, for 6.25 μg/mL and 25 μg/mL) are expressed as dot plots, along with the mean ± SD. To promote their visualization, the results for different treatment concentrations are spaced evenly along the X‐axis, without regard to relative concentration. *p < .05 was considered statistically significant compared to the concurrent vehicle control (one‐tailed Dunnett's test). Primary data for the plot shown in this figure are reported in Table 1

The frequency of single‐nucleotide mutations was measured directly with ecNGS Duplex Sequencing using DNA extracted from cultures treated with EMS for 28 days. EMS treatment resulted in a concentration‐dependent increase in the per‐nucleotide mutant frequency (slope of linear regression line was significantly non‐zero, *p* = .0069, Figure 6). The mean mutant frequencies measured by Duplex Sequencing in the negative control and the cultures treated with increasing concentrations of EMS were 1.9, 2.3, 3.1, 4.0, and 10.3 × 10^−7^ (Figure 6 and Table 1). Compared with the vehicle control, significant increases were observed in cultures treated with 25, 50, and 100 μg/mL of EMS.

**FIGURE 7:**
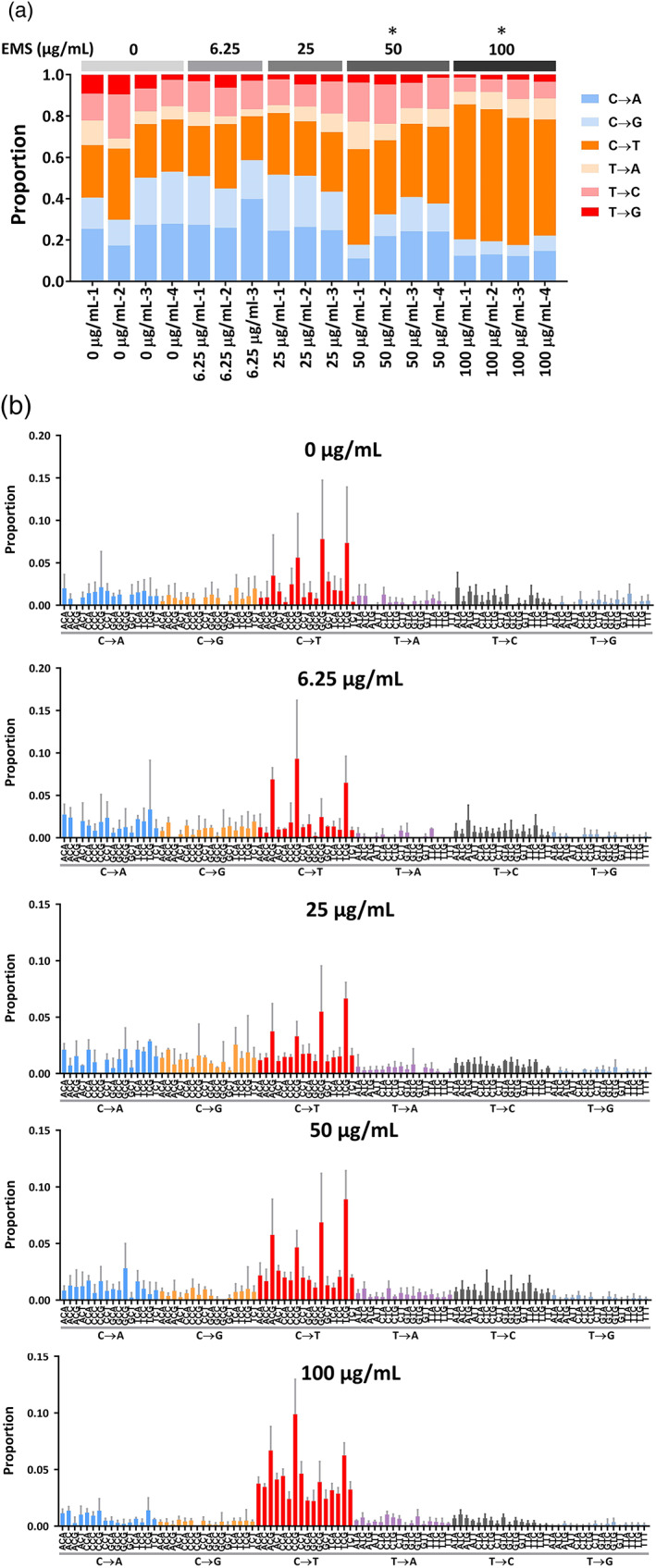
Mutation spectra analysis. (a) Proportions of simple base substitutions are plotted for each sample. *p < .05 was considered statistically significant differences in the proportion of C → T transitions, when compared to the concurrent vehicle control. (b) Trinucleotide spectra of treatment groups show distinct patterns of mutagenesis specific to EMS treatment

Mutant spectra often can reveal mechanistic information on the nature of a mutagen and the molecular processes involved in mutagenesis and DNA repair. Simple base‐substitution spectra (Figure 7(a)) show that the proportion of pyrimidine‐normalized C → T transitions increased with increasing concentrations of EMS, with significant increases observed in cultures exposed to the two highest concentrations of EMS: 50 and 100 μg/mL. Gross visualization of the Duplex Sequencing data shows that the increase in C → T transitions in samples exposed to the two highest concentrations of EMS occurred across all trinucleotide contexts, in contrast to the DMSO control data in which C → T transitions cluster in CpG motifs (Figure 7(b)). The control pattern is likely the result of age‐associated spontaneous deamination of 5‐methylcytosine residues (Alexandrov et al., 2015; Valentine et al., 2020).

## 4 DISCUSSION

This study integrated the measurement of mutagenesis and DNA damage into the evaluation of toxicity and tissue function in organotypic human in vitro airway tissue cultures. The high concentration of EMS used for the study (100 μg/mL) was mildly cytotoxic, at least at some sampling times, and resulted in a reduced abundance of anti‐Ki67‐stained proliferating cells. The 100 μg/mL concentration of EMS also inhibited CBF and mucin secretion, indicating an attenuation of MCC. Lower concentrations of EMS had only minor effects on these endpoints. We conclude that the concentration range we used for this proof‐of‐principle assay of genetic toxicology endpoints achieved a maximum concentration tolerated by the system. The overlapping concentration ranges for general toxicity and genotoxicity endpoints allowed us to integrate both into a single efficient study.

It should be noted that this exploratory study used exposure to EMS in the basal medium. While this could be argued as an appropriate route for mimicking systemic exposure of the tissue model, this was done mostly for convenience and enabled us to concentrate our efforts on whether or not the genetic toxicology endpoints could be measured in these cultures. One of the unique attributes of these tissue models is their appropriate cell polarity and air interface. The air interface of the airway epithelial tissue model facilitates direct exposure to gases, aerosols, particulates, cigarette smoke, etc., in a manner similar to in vivo inhalation exposure conditions. Studies involving apical exposures could provide data that are more valuable for the toxicological evaluation of inhaled substances (Cao et al., 2020).

The CometChip data indicated that all concentrations of EMS significantly damaged the DNA of the tissue models and that the damage manifested in a time‐ and concentration‐dependent manner. It is interesting that the DNA strand breaks and alkali‐labile sites measured by the CometChip assay seemed to accumulate with time, even at the lowest concentration of EMS, suggesting that the DNA repair mechanisms present in the slowly dividing cultures were insufficient to maintain the integrity of the DNA during the repeat‐treatment protocol used for this study. It should be kept in mind, however, that much of this damage may be irrelevant for mutation induction. A minority of the cells proliferate in the culture during natural cell turnover and in response to injury; also, the type of damage measured by the CometChip assay may not be the damage that results in the mutations evaluated by Duplex Sequencing. Thus, CometChip measurements are simply one estimate of the level of DNA damage in the cultures and may not correlate directly with the burden of mutations ultimately fixed in the genome.

We observed that a substantial amount of DNA damage was present in the DNA of the tissue models after the 28‐day exposure period. Thus, it is possible that increasing the mutation fixation time by sampling after a recovery period of 2 or 4 weeks might show a further increased mutation burden. This option is recommended for detecting mutation in slowly dividing tissues (e.g. liver) in the transgenic rodent gene mutation assay (OECD, 2020), and it may have a similar effect on mutation frequencies in these slowly dividing cell cultures. This speculation remains to be tested empirically.

It has long been held that the majority of mutations induced by exposure to chemicals and radiation requires replication of mutagen‐damaged cells and that any DNA repair that occurs in quiescent cells before cell replication reduces the frequency of mutation induction (Konze‐Thomas et al., 1982; Yang et al., 1982). These classical studies were conducted with synchronized cultures of replicating cells treated at different stages of the cell cycle. Fully differentiated human airway tissue cultures represent a dynamic system consisting of different cell types with different proliferative capacities, including many fully differentiated cells having little proliferative capacity. Models might be constructed with higher proliferation rates, presumably resulting in more efficient mutation fixation, but such models would sacrifice their functional similarity to in vivo tissue. The major proliferative cell type found in human airway tissue models is the basal cell, which makes up approximately 30% to 40% of the total cells (Boers et al., 1998; Cao et al., 2020) (Figure 4(b)). As basal cells self‐renew and differentiate to replenish other epithelial cell populations, including the ciliated cells and secretory goblet cells, basal cell proliferation is likely to be one of the key determinants in the portion of cells mutagenized in the airway tissue culture. It might be possible to select for basal cells via histochemical enrichment and focus the mutation analysis on this cell population. During a 28‐day treatment, the cells transiting from the anti‐Ki67‐positive basal cell population to the ciliated or goblet cell populations may have been small; but, because of the extended treatment protocol, they also could make a significant contribution to the portion of mutagenized cells in the cultures.

Given that the majority of cells in the airway model are fully differentiated and are presumably non‐dividing, it may be appropriate to consider if DNA synthesis in non‐replicating cells could be responsible for a portion of the EMS‐induced mutations in the airway cultures. Error‐prone DNA synthesis may be enhanced by the need to repair adducts and other forms of DNA damage such as strand breaks in EMS‐treated cultures. Because of the extended treatment times in our study, such sources of mutation induction, albeit inefficient, might be significant for mutation induction in the airway tissue model. Evaluating mutations in specific cell populations, as indicated above, might shed light on this potential source of mutations. For example, if cell replication was the source of EMS‐induced mutations, the expectation is that mutations would appear first in cells enriched for Ki67 expression; if repair was a source of mutations, mutations might be expected to appear early in both replicating and non‐replicating cell populations.

Mutation induction in dividing and non‐dividing cells, described above, both result in true mutations. But as Duplex Sequencing is a relatively new approach for evaluating mutagenesis, it is conceivable that the mutations detected by Duplex Sequencing could be an artifact of analyzing a damaged DNA template. However, at least two unpublished data sets from studies conducted with *N*‐ethyl‐*N*‐nitrosourea (ENU) make such a source of mutations unlikely. Cho, Yauk and colleagues reported that Duplex Sequencing, performed shortly after treating human TK6 cells with ENU, failed to detect an increase in mutations, yet identified a clear induction 48 h after the initial exposure (Carole Yauk, personal communication). Smith‐Roe, Hobbs, Witt and colleagues reported that mutation induction could be measured in bone marrow as early as 24 h following in vivo dosing of rats with ENU. But it took up to 7 days to detect induced mutations in the more slowly dividing liver, despite all tissues being exposed to the rapidly distributed mutagen at the same time (Smith‐Roe et al., 2020). These observations are particularly relevant to our in vitro study because ENU produces a spectrum of DNA damage similar to that produced by EMS (Beranek et al., 1980; Heflich et al., 1982).

EMS is an ethylating agent that is mutagenic in a variety of genetic test systems from bacteria, to in vitro cell lines, to in vivo animal models (Beranek, 1990; Saini et al., 2020; Sega, 1984). EMS alkylates nucleophilic sites in DNA through a mixed SN1/SN2 reaction mechanism (Sega, 1984). While ethylation of DNA occurs principally at nitrogen positions in the bases and forms N‐7‐alkylguanine, EMS also is able to produce significant levels of alkylation at oxygens such as the *O* ^6^ of guanine and in the DNA phosphate groups which may be a cause for at least part of the mutagenic activity of EMS (Beranek, 1980; Loechler et al., 1984; Saini et al., 2020; Shrivastav et al., 2010). Genetic data from different biological systems suggest that EMS commonly leads to C → T changes in DNA (Olivier et al., 2014; Sega, 1984; Westcott et al., 2015). In agreement with these findings, the mutations detected by Duplex Sequencing in DNA from EMS‐treated airway models demonstrated a concentration‐dependent increase in C → T changes, indicating that in vitro organotypic airway cultures are capable of providing mutation signatures typical of DNA alkylating agents.

The three highest concentrations of EMS (25, 50 and 100 μg/mL) all produced significant increases in mutant frequency relative to the negative control, and a significant linear relationship between increasing mutant frequency and increasing EMS concentration was observed. Thus, a no‐effect threshold was not clearly present for EMS treatment in our study (for either mutation or DNA damage). This is in contrast to studies conducted in human cell lines and in transgenic mice (Cao et al., 2014; Doak et al., 2007; Müller et al., 2009), where EMS treatment resulted in no‐observed‐effect shoulders on the mutation concentration responses. It is also of note that, in a study conducted previously in *gpt*‐delta mice, whole lung tissue was more sensitive to EMS mutagenicity and had lower points of departure than was seen in other tissues (Cao et al., 2014). These observations lead us to speculate that lung tissue may be particularly sensitive to EMS‐induced mutagenicity.

Although there was no direct indication of a “rebound” associated with the toxicity associated with anti‐Ki67‐positive proliferating cells in this study (e.g., cell loss due to toxicity followed by compensatory cell proliferation), there is a possibility that alterations to cell proliferation caused by the treatment can affect, and increase, mutation frequencies in ALI airway cultures. Such an increase in anti‐Ki67‐staining cells was seen, for instance, in human airway cultures treated with the chemical sterilant, ortho‐phthalaldehyde (Wang et al., 2021). ALI cultures are known to adapt to toxic insults, with toxicity and cell loss followed by compensatory cell proliferation and tissue repair (Cao et al., 2020; Wang et al., 2021; Xiong et al., 2018). Such an adaptation may account for our observation of an increase in LDH release at Day 7 in cultures treated with the high concentration of EMS, followed by a decrease in LDH release as the number of treatments increased (Figure 2). The extent to which alterations in tissue proliferation and differentiation kinetics can contribute to mutant frequencies in these models (and potentially in vivo) bears further investigation.

In summary, we have demonstrated that increases in DNA damage and mutation can be detected in human in vitro ALI airway tissue models treated with the known mutagen, EMS. In particular, ecNGS Duplex Sequencing was able to overcome the challenges involved in measuring mutation in these highly differentiated organotypic tissue models. However, a substantial amount of information regarding the nature of these responses is yet to be learned. Because the CometChip and Duplex Sequencing assays can be conducted with limited numbers of cells from highly differentiated organotypic models, it is likely that these approaches can be used with other tissue and cell models, such as the skin ALI model which is being used for comet and micronucleus evaluation (Curren et al., 2006; Reus et al., 2013), epithelial cell spheroids (Guo et al., 2020), and more complex emerging microphysiological systems.

## CONFLICT OF INTEREST

E.K.S., T.H.S., Z.K.N., C.C.V., J.F., L.N.W., and J.J.S. are employees and equity holders at TwinStrand Biosciences, Inc. E.K.S., C.C.V., L.N.W., and J.J.S. are authors on one or more Duplex Sequencing‐related patents.

## AUTHOR CONTRIBUTIONS

Yiying Wang—study director, participated in every element of the study. Roberta A. Mittelstaedt—exposure of ALI airway cultures to EMS, LDH cytotoxicity assay, CBF measurement. Rebecca Wynne—CometChip assay. Ying Chen—genomic DNA extraction. Xuefei Cao—human ALI airway tissue models and concept. Levan Muskhelishvili and Kelly Davis—histology, immunohistochemistry and image analysis. Elizabeth K. Schmidt, Thomas H. Smith, Zachary K. Norgaard, Charles C. Valentine, Jeffry Yaplee, Lindsey N. Williams, and Jesse J. Salk—Duplex Sequencing. Timothy W. Robison and Wei Sun—concept formulation. Robert H. Heflich—concept, study design, and manuscript writing. All authors had input in writing the manuscript and approved the submitted version.

## DISCLAIMER

The findings and conclusions in this report are those of the authors and do not necessarily represent those of U.S. Food and Drug Administration positions or policies. The mention of commercial products, their sources, or their use in connection with material reported herein is not to be construed as either an actual or implied endorsement of such products.

## Supporting information


**Table S1** Measurement of lactate dehydrogenase (LDH) release into the basolateral medium.
**Table S2**. Measurement of cilia beating frequency (CBF) over a 28‐day exposure.
**Table S3**. Measurement of MUC5AC secretion on Day 14 and Day 28.
**Table S4**. Measurement of MUC5B secretion on Day 14 and Day 28.
**Table S5**. Quantification of the percentage of anti‐Ki67, anti‐p63‐positive cells and goblet cells.
**Table S6**. Measurement of DNA damage using the CometChip assay after 3‐day and 28‐day treatments.
**Table S7**. Measurement of the relative cell viability (% of control) using the MTS assay after 3‐day and 28‐day treatments.

## Data Availability

The data underlying this article are available in the article and in its online supplementary material.
