# Supplementary material for: Genetic toxicity testing using human in vitro organotypic airway cultures: Assessing DNA damage with the CometChip and mutagenesis by Duplex Sequencing
Source: Environ Mol Mutagen. 2021 Jun 14;62(5):306–18. doi: 10.1002/em.22444 (PMC8251634; doi:10.1002/em.22444)
Supplement: Supplementary file 1 — Table S1 Measurement of lactate dehydrogenase (LDH) release into the basolateral medium. Table S2. Measurement of cilia beating frequency (CBF) over a 28‐day exposure. Table S3. Measurement of MUC5AC secretion on Day 14 and Day 28. Table S4. Measurement of MUC5B secretion on Day 14 and Day 28. Table S5. Quantification of the percentage of anti‐Ki67, anti‐p63‐positive cells and goblet cells. Table S6. Measurement of DNA damage using the CometChip assay after 3‐day and 28‐day treatments. Table S7. Measurement of the relative cell viability (% of control) using the MTS assay after 3‐day and 28‐day treatments. [file EM-62-306-s001.docx]

**Supplementary Material**

**Supplementary Table 1**. Measurement of lactate dehydrogenase (LDH) release into the basolateral medium

| **EMS µg/ml** | **OD_490 nm_ (D1)** | | | | **AVE** | **SD** |
| --- | --- | --- | --- | --- | --- | --- |
|  | **1** | **2** | **3** | **4** |  |  |
| 0 | 0.032 | 0.040 | 0.038 | 0.041 | 0.038 | 0.003 |
| 6.25 | 0.046 | 0.048 | 0.059 | 0.068 | 0.055 | 0.009 |
| 25 | 0.057 | 0.053 | 0.059 | 0.068 | 0.059 | 0.005 |
| 50 | 0.056 | 0.045 | 0.059 | 0.061 | 0.055 | 0.006 |
| 100 | 0.058 | 0.067 | 0.067 | 0.061 | 0.063 | 0.004 |
| **EMS µg/ml** | **OD_490 nm_ (D7)** | | | | **AVE** | **SD** |
|  | **1** | **2** | **3** | **4** |  |  |
| 0 | 0.162 | 0.173 | 0.144 | 0.157 | 0.159 | 0.010 |
| 6.25 | 0.143 | 0.136 | 0.177 | 0.155 | 0.138 | 0.014 |
| 25 | 0.148 | 0.125 | 0.132 | 0.116 | 0.130 | 0.012 |
| 50 | 0.138 | 0.146 | 0.140 | 0.168 | 0.148 | 0.012 |
| 100 | 0.489 | 0.472 | 0.481 | 0.497 | 0.485 | 0.011 |
| **EMS µg/ml** | **OD_490 nm_ (D14)** | | | | **AVE** | **SD** |
|  | **1** | **2** | **3** | **4** |  |  |
| 0 | 0.103 | 0.098 | 0.078 | 0.137 | 0.104 | 0.021 |
| 6.25 | 0.072 | 0.076 | 0.066 | 0.109 | 0.081 | 0.017 |
| 25 | 0.084 | 0.077 | 0.079 | 0.110 | 0.088 | 0.013 |
| 50 | 0.099 | 0.096 | 0.091 | 0.104 | 0.098 | 0.005 |
| 100 | 0.187 | 0.192 | 0.181 | 0.221 | 0.195 | 0.015 |
| **EMS µg/ml** | **OD_490 nm_ (D21)** | | | | **AVE** | **SD** |
|  | **1** | **2** | **3** | **4** |  |  |
| 0 | 0.078 | 0.082 | 0.071 | 0.083 | 0.079 | 0.004 |
| 6.25 | 0.069 | 0.071 | 0.067 | 0.083 | 0.072 | 0.006 |
| 25 | 0.075 | 0.062 | 0.062 | 0.063 | 0.066 | 0.006 |
| 50 | 0.086 | 0.090 | 0.072 | 0.089 | 0.084 | 0.007 |
| 100 | 0.126 | 0.111 | 0.109 | 0.124 | 0.118 | 0.008 |
| **EMS µg/ml** | **OD_490 nm_ (D28)** | | | | **AVE** | **SD** |
|  | **1** | **2** | **3** | **4** |  |  |
| 0 | 0.065 | 0.056 | 0.052 | 0.070 | 0.061 | 0.007 |
| 6.25 | 0.052 | 0.057 | 0.058 | 0.087 | 0.064 | 0.014 |
| 25 | 0.062 | 0.052 | 0.049 | 0.057 | 0.055 | 0.005 |
| 50 | 0.051 | 0043 | 0.050 | 0.076 | 0.055 | 0.012 |
| 100 | 0.069 | 0.061 | 0.053 | 0.157 | 0.052 | 0.042 |

EMS, Ethyl methanesulfonate; SD, Standard Deviation; D1, Day 1; D7, Day 7, etc.

**Supplementary Table 2.** Measurement of cilia beating frequency (CBF) over a 28-day exposure

| **EMS µg/ml** | **CBF Hz (D1)** | | | | **AVE** | **SD** |
| --- | --- | --- | --- | --- | --- | --- |
|  | **1** | **2** | **3** | **4** |  |  |
| 0 | 5.506 | 5.500 | 4.604 | 5.474 | 5.271 | 0.445 |
| 6.25 | 4.230 | 4.757 | 4.775 | 5.628 | 4.848 | 0.578 |
| 25 | 5.633 | 5.333 | 6.118 | 5.740 | 5.706 | 0.324 |
| 50 | 4.553 | 4.243 | 5.224 | 3.686 | 4.426 | 0.642 |
| 100 | 3.763 | 5.105 | 3.903 | 4.115 | 4.228 | 0.602 |
| **EMS µg/ml** | **CBF Hz (D7)** | | | | **AVE** | **SD** |
|  | **1** | **2** | **3** | **4** |  |  |
| 0 | 6.800 | 6.390 | 6.350 | 5.800 | 6.335 | 0.410 |
| 6.25 | 6.173 | 5.732 | 5.674 | 5.595 | 5.794 | 0.259 |
| 25 | 5.652 | 5.524 | 5.324 | 5.923 | 5.606 | 0.251 |
| 50 | 4.688 | 5.110 | 4.594 | 5.923 | 4.806 | 0.225 |
| 100 | 4.393 | 4.032 | 4.705 | 4.743 | 4.468 | 0.330 |
| **EMS µg/ml** | **CBF Hz (D14)** | | | | **AVE** | **SD** |
|  | **1** | **2** | **3** | **4** |  |  |
| 0 | 6.803 | 6.050 | 5.904 | 5.588 | 6.086 | 0.515 |
| 6.25 | 5.864 | 5.936 | 5.450 | 6.100 | 5.838 | 0.277 |
| 25 | 5.393 | 5.434 | 5.680 | 5.925 | 5.608 | 0.246 |
| 50 | 4.318 | 4.267 | 4.696 | 4.190 | 4.368 | 0.225 |
| 100 | 3.292 | 3.155 | 3.173 | 3.148 | 3.192 | 0.067 |
| **EMS µg/ml** | **CBF Hz (D21)** | | | | **AVE** | **SD** |
|  | **1** | **2** | **3** | **4** |  |  |
| 0 | 6.208 | 7.338 | 5.973 | 6.284 | 6.451 | 0.606 |
| 6.25 | 5.676 | 6.056 | 6.604 | 5.714 | 6.012 | 0.430 |
| 25 | 4.863 | 7.348 | 6.210 | 5.960 | 6.095 | 1.120 |
| 50 | 4.995 | 3.770 | 4.132 | 4.860 | 4.439 | 0.585 |
| 100 | N.D | N.D | N.D | N.D | N.D | N.D |
| **EMS µg/ml** | **CBF Hz (D28)** | | | | **AVE** | **SD** |
|  | **1** | **2** | **3** | **4** |  |  |
| 0 | 6.045 | 6.408 | 5.598 | 4.947 | 5.70 | 0.629 |
| 6.25 | 6.396 | 6.580 | 6.103 | 6.142 | 6.305 | 0.224 |
| 25 | 6.453 | 5.785 | 5.960 | 5.980 | 6.044 | 0.286 |
| 50 | 4.713 | 4.374 | 4.496 | 4.360 | 4.486 | 0.163 |
| 100 | N.D | N.D | N.D | N.D | N.D | N.D |

EMS, Ethyl methanesulfonate; SD, Standard Deviation; D1, Day 1; D7, Day 7, etc.; N.D: not detected.

**Supplementary Table 3.** Measurement of MUC5AC secretion on Day 14 and Day 28

| **EMS µg/ml** | **OD_450 nm_ (D14)** | | | | **AVE** | **SD** |
| --- | --- | --- | --- | --- | --- | --- |
|  | **1** | **2** | **3** | **4** |  |  |
| 0 | 0.576 | 0.681 | 0.889 | 0.701 | 0.712 | 0.130 |
| 6.25 | 0.822 | 0.734 | 0.862 | 0.737 | 0.789 | 0.064 |
| 25 | 0.770 | 0.736 | 0.654 | 0.716 | 0.716 | 0.049 |
| 50 | 0.558 | 0.554 | 0.542 | 0.532 | 0.546 | 0.012 |
| 100 | 0.439 | 0.442 | 0.473 | 0.481 | 0.459 | 0.021 |
| **EMS µg/ml** | **OD_450 nm_ (D28)** | | | | **AVE** | **SD** |
|  | **1** | **2** | **3** | **4** |  |  |
| 0 | 0.684 | 0.786 | 0.709 | 0.579 | 0.690 | 0.086 |
| 6.25 | 0.639 | 0.710 | 0.785 | 0.592 | 0.682 | 0.084 |
| 25 | 0.681 | 0.611 | 0.609 | 0.604 | 0.626 | 0.037 |
| 50 | 0.334 | 0.388 | 0.341 | 0.354 | 0.354 | 0.024 |
| 100 | 0.250 | 0.166 | 0.216 | 0.229 | 0.215 | 0.036 |

EMS, Ethyl methanesulfonate; SD, Standard Deviation; D14, Day 14; D28, Day 28.

**Supplementary Table 4.** Measurement of MUC5B secretion on Day 14 and Day 28

| **EMS µg/ml** | **OD_450 nm_ (D14)** | | | | **AVE** | **SD** |
| --- | --- | --- | --- | --- | --- | --- |
|  | **1** | **2** | **3** | **4** |  |  |
| 0 | 0.109 | 0.126 | 0.123 | 0.106 | 0.116 | 0.010 |
| 6.25 | 0.201 | 0.121 | 0.081 | 0.112 | 0.129 | 0.051 |
| 25 | 0.108 | 0.067 | 0.100 | 0.090 | 0.087 | 0.022 |
| 50 | 0.119 | 0.080 | 0.083 | 0.067 | 0.087 | 0.022 |
| 100 | 0.088 | 0.086 | 0.091 | 0.136 | 0.100 | 0.024 |
| **EMS µg/ml** | **OD_450 nm_ (D28)** | | | | **AVE** | **SD** |
|  | **1** | **2** | **3** | **4** |  |  |
| 0 | 0.125 | 0.124 | 0.145 | 0.125 | 0.130 | 0.010 |
| 6.25 | 0.199 | 0.156 | 0.131 | 0.117 | 0.131 | 0.018 |
| 25 | 0.129 | 0.133 | 0.111 | 0.121 | 0.124 | 0.010 |
| 50 | 0.106 | 0.082 | 0.090 | 0.107 | 0.096 | 0.012 |
| 100 | 0.063 | 0.083 | 0.086 | 0.061 | 0.074 | 0.013 |

EMS, Ethyl methanesulfonate; SD, Standard Deviation; D14, Day 14; D28, Day 28.

**Supplementary Table 5.** Quantification of the percentage of anti-Ki67, anti-p63-positive cells and goblet cells

| **Anti-Ki67 density (%)** | | | | | |
| --- | --- | --- | --- | --- | --- |
| **EMS µg/ml** | **1** | **2** | **3** | **AVE** | **SD** |
| 0 | 6.8 | 5.5 | 4.8 | 5.7 | 1.0 |
| 25 | 5.6 | 7.2 | 5.7 | 6.2 | 0.9 |
| 100 | 2.0 | 1.8 | 3.9 | 2.6 | 1.2 |
| **Anti-p63 density (%)** | | | | | |
| **EMS µg/ml** | **1** | **2** | **3** | **AVE** | **SD** |
| 0 | 38.5 | 37.0 | 36.0 | 37.2 | 1.3 |
| 25 | 36.5 | 30.0 | 30.9 | 32.5 | 3.5 |
| 100 | 38.0 | 35.2 | 34.1 | 35.8 | 2.0 |
| **Goblet Cell density (%)** | | | | | |
| **EMS µg/ml** | **1** | **2** | **3** | **AVE** | **SD** |
| 0 | 20.9 | 24.8 | 27.7 | 24.5 | 3.4 |
| 25 | 41.8 | 31.1 | 38.0 | 37.0 | 5.4 |
| 100 | 2.9 | 5.4 | 2.0 | 3.4 | 1.8 |

EMS, Ethyl methanesulfonate; SD, Standard Deviation.

**Supplementary Table 6.** Measurement of DNA damage using the CometChip assay after 3-day and 28-day treatments

| **CometChip** | | | | | | |
| --- | --- | --- | --- | --- | --- | --- |
| **EMS µg/ml** | **%DNA in Tail (D3)** | | | | **AVE** | **SD** |
|  | **1** | **2** | **3** | **4** |  |  |
| 0 | 4.83 | 4.41 | 2.44 | 3.63 | 3.83 | 1.05 |
| 6.25 | 6.86 | 11.92 | 11.97 | 10.03 | 10.20 | 2.40 |
| 25 | 29.48 | 28.16 | 23.33 | 33.71 | 28.67 | 4.28 |
| 50 | 53.54 | 60.67 | 58.63 | 61.13 | 58.49 | 3.48 |
| 100 | 87.81 | 77.96 | 86.37 | 81.06 | 83.30 | 4.59 |
| **EMS µg/ml** | **%DNA in Tail (D28)** | | | | **AVE** | **SD** |
|  | **1** | **2** | **3** | **4** |  |  |
| 0 | 7.54 | 4.19 | 8.6 | 6.52 | 6.71 | 1.88 |
| 6.25 | 37.39 | 32.7 | 26.87 | 36.87 | 33.46 | 4.87 |
| 25 | 83.12 | 76.44 | 73.99 | 80.32 | 78.47 | 4.05 |
| 50 | 85.76 | 92.95 | 85.97 | 83.19 | 86.97 | 4.18 |
| 100 | 86.35 | 85.41 | 87.17 | 90.03 | 87.24 | 1.99 |

EMS, Ethyl methanesulfonate; SD, Standard Deviation; D3, Day 3; D28, Day 28.

**Supplementary Table 7.** Measurement of the relative cell viability (% of control) using the MTS assay after 3-day and 28-day treatments

| **MTS** | | | | | | |
| --- | --- | --- | --- | --- | --- | --- |
| **EMS µg/ml** | **OD_490 nm_ (D3)** | | | | **AVE** | **SD** |
|  | **1** | **2** | **3** | **4** |  |  |
| 0 | 0.201 | 0.219 | 0.210 | 0.198 | 0.207 | 0.010 |
| 6.25 | 0.168 | 0.199 | 0.193 | 0.197 | 0.189 | 0.014 |
| 25 | 0.178 | 0.223 | 0.227 | 0.223 | 0.213 | 0.023 |
| 50 | 0.189 | 0.197 | 0.199 | 0.201 | 0.196 | 0.005 |
| 100 | 0.195 | 0.223 | 0.204 | 0.214 | 0.209 | 0.012 |
| **EMS µg/ml** | **OD_490 nm_ (D28)** | | | | **AVE** | **SD** |
|  | **1** | **2** | **3** | **4** |  |  |
| 0 | 0.224 | 0.249 | 0.237 | 0.271 | 0.245 | 0.020 |
| 6.25 | 0.211 | 0.285 | 0.231 | 0.218 | 0.236 | 0.033 |
| 25 | 0.210 | 0.259 | 0.273 | 0.212 | 0.239 | 0.032 |
| 50 | 0.242 | 0.279 | 0.220 | 0.239 | 0.245 | 0.025 |
| 100 | 0.192 | 0.210 | 0.192 | 0.183 | 0.194 | 0.011 |

EMS, Ethyl methanesulfonate; MTS, 3-(4,5-dimethylthiazol-2-yl)-5-(3-carboxymethoxyphenyl)-2-(4-sulfophenyl)-2H-tetrazolium; SD, Standard Deviation; D3, Day e; D28, Day 28.
